# Maternal Folic Acid Supplementation and Childhood Metabolic Health: Analyzing Direct and Indirect Effects

**DOI:** 10.1002/fsn3.70959

**Published:** 2025-09-16

**Authors:** Mehak Batra, Yibeltal Bekele, Eva Karaglani, Yannis Manios, George Moschonis, Bircan Erbas

**Affiliations:** ^1^ Department of Public Health, School of Psychology and Public Health La Trobe University Melbourne Victoria Australia; ^2^ Department of Reproductive Health, School of Public Health Bahir Dar University Bahir Dar Ethiopia; ^3^ Department of Nutrition and Dietetics, School of Health Science and Education Harokopio University Athens Greece; ^4^ European Centre for Obesity Research Harokopio University Athens Greece; ^5^ Institute of Agri‐Food and Life Sciences, Hellenic Mediterranean University Research Centre Crete Greece; ^6^ Department of Food, Nutrition and Dietetics, School of Allied Health, Human Services and Sport La Trobe University Bundoora Victoria Australia; ^7^ La Trobe Institute for Sustainable Agriculture and Food (LISAF), La Trobe University Melbourne Australia

**Keywords:** childhood metabolic, folic acid, obesity markers, perinatal epidemiology

## Abstract

This study examines the direct and indirect effects of maternal folic acid (FA) supplementation on childhood metabolic outcomes, including blood pressure (BP), insulin resistance (HOMA‐IR), and insulin sensitivity (QUICKI), with body mass index (BMI), waist circumference (WC), and skinfold thickness as mediators. This cross‐sectional study analyzed data from 2577 children (9–13 years) from the Healthy Growth Study (2007–2009, Greece). Maternal FA intake was reported via parental questionnaires. BMI, WC, and skinfold thickness were measured using standardized protocols. BP, fasting glucose, and insulin were assessed to compute HOMA‐IR and QUICKI. Structural equation modeling evaluated direct and indirect effects, adjusting for maternal, pregnancy, and child‐related confounders. FA supplementation had no direct effect. However, indirect effects via WC were associated with lower SBP (*β* = −0.69, *p* = 0.036) and DBP (*β* = −0.36, *p* = 0.041), improved insulin sensitivity (QUICKI: *β* = 0.0018, *p* = 0.044), and reduced insulin resistance (HOMA‐IR: *β* = −0.1099, *p* = 0.047). Skinfold thickness also mediated BP reductions. BMI was not a significant mediator (all *p* > 0.05). Maternal FA intake may indirectly influence childhood metabolic health via adiposity markers, emphasizing the need for targeted maternal nutrition interventions to improve childhood metabolic outcomes.

## Graphical abstract

First study to demonstrate that folic acid supplementation indirectly influences childhood blood pressure and insulin sensitivity via waist circumference and skinfold thickness. BMI was not a mediator, suggesting that specific fat distribution patterns, rather than overall adiposity, may be more relevant to metabolic outcomes. Recommendation: Waist circumference and skinfold thickness are modifiable adiposity markers and present actionable targets for public health strategies aimed at improving long‐term metabolic health.

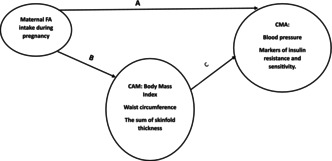

## 1 Introduction

Maternal nutrition during pregnancy is a critical determinant of long‐term health outcomes in offspring. While folic acid (FA) is widely recognized for preventing neural tube defects, emerging evidence indicates its potential influence on fetal metabolic programming and consequently childhood metabolic health (Pereira and Keating 2023). However, the mechanistic pathways through which maternal FA intake during pregnancy impacts offspring metabolic outcomes, such as insulin sensitivity and blood pressure (BP), remain underexplored, particularly during late childhood.

One‐carbon metabolism, regulated by FA, governs DNA methylation and gene expression, which are crucial for fetal development and metabolic programming (Padmanabhan and Watson 2013). These processes are hypothesized to influence adiposity markers, such as body mass index (BMI), waist circumference (WC), and skinfold thickness, which are strongly linked to metabolic outcomes like insulin resistance and hypertension (González‐Torres et al. 2023; Feng et al. 2012). However, most existing studies focus on general associations without delving into the biological pathways or mediators.

Moreover, current evidence is often limited to early childhood or populations with mandatory FA fortification, leaving gaps in understanding the long‐term metabolic implications in diverse contexts. Few studies have employed advanced statistical techniques, such as structural equation modeling (SEM), to disentangle FA intake's direct and indirect effects through adiposity markers. This methodological gap limits our ability to identify modifiable intervention factors for reducing the burden of metabolic diseases.

This brief communication addresses these gaps by examining the mediating role of specific adiposity markers in the relationship between maternal FA supplementation and childhood metabolic outcomes in late childhood. By providing robust insights into these pathways, we aim to inform targeted maternal nutrition strategies and contribute to the prevention of metabolic disorders in children.

## 2 Methods

This cross‐sectional study used data from the Healthy Growth Study (2007–2009), including 2577 schoolchildren (aged 9–13 years) from 77 schools across Greece. Ethical approval was granted by the Greek Ministry of National Education and Harokopio University of Athens Ethics Committee, following the Declaration of Helsinki. A multistage stratified random sampling method ensured representativeness (response rate: 64.1%), and written parental consent was obtained. Sociodemographic and perinatal data were collected via standardized parental interviews and validated with birth certificates and medical records.

### 2.1 Primary Exposure Variable

A validated parental questionnaire assessed FA intake during pregnancy, including trimester‐specific supplementation timing.

### 2.2 Mediators

Two trained researchers used consistent instruments and procedures to conduct standardized measurements of body weight, height, BMI, and WC.

### 2.3 Primary Outcome Variables

#### 2.3.1 Blood Pressure Assessment

BP was measured using an electronic BP monitor at the right brachial artery after 5‐min rest. Two readings (averaged) were taken 2 min apart, with a third measurement if readings differed by > 10 mmHg.

#### 2.3.2 Biochemical Analyses

Fasting morning blood samples (12‐h fast) were collected, with plasma glucose measured via enzymatic colorimetric assays (Roche Diagnostics SA) and serum insulin via chemiluminescence immunoassay (Kyowa Medex Ltd). Insulin resistance (HOMA‐IR) and insulin sensitivity (QUICKI) indices were calculated:

- HOMA‐IR: (Fasting Insulin × Fasting Glucose)/22.5
- QUICKI: 1/[log(Fasting Insulin) + log(Fasting Glucose)]

Both indices are validated for assessing insulin resistance and sensitivity in non‐diabetic children (Manios et al. 2014).

### 2.4 Statistical Analysis

Descriptive statistics were calculated to summarize the characteristics of the study population, stratified by maternal FA intake during pregnancy. Continuous variables were reported as mean ± standard deviation (SD) and median with interquartile range (IQR), while categorical variables were presented as frequencies and percentages. Differences between groups were assessed using the Mann–Whitney *U* test for continuous variables (due to non‐normal distribution) and the chi‐squared test for categorical variables.

The data were analyzed using SEM to evaluate the direct and indirect effects of prenatal FA intake on metabolic outcomes in offspring. The SEM approach allows for the simultaneous estimation of multiple regression equations, comprehensively analyzing the hypothesized pathways and mediators. The SEM included pathways from FA intake to metabolic outcomes (QUICKI, HOMA‐IR) directly and through mediators (the sum of skinfold thickness, BMI, and WC). The maximum likelihood (ML) estimation method was used with 1000 bootstrap replications to ensure robust standard errors and confidence intervals. Control variables included pregnancy‐related factors (gestational diabetes mellitus [GDM], high blood pressure [HBP] during pregnancy), infant feeding practices (exclusive breastfeeding duration), maternal and pregnancy characteristics (conception timing, maternal smoking during pregnancy, parity, maternal age, maternal education), demographic factors (race, pregnancy weight gain, small for gestational age), and child‐specific factors (gender, total caloric intake, moderate to vigorous physical activity [MVPA]).

**FIGURE 1:**
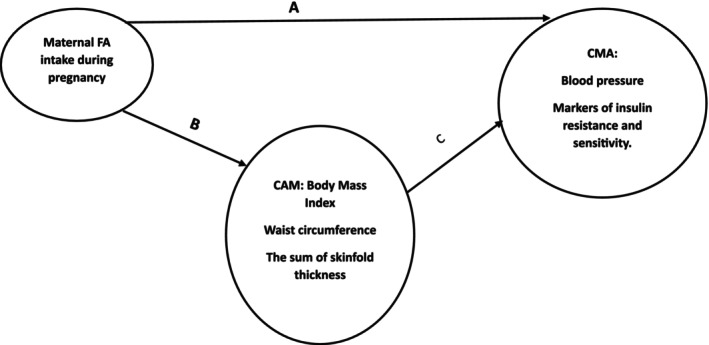
The direct and indirect relationship between maternal folic acid supplementation and childhood metabolic health (9–13 years). A = direct effect (FA → CMH); B = FA → CAM; C = CAM → CMH; indirect effect = B × C. CAM, childhood adiposity markers; CMH, childhood metabolic health; FA, folic acid.

The indirect effects were estimated to understand the mediation pathways. For instance, the indirect effect of FA intake on metabolic outcomes through the sum of skinfold thickness was calculated (Figure 1). Model fit was evaluated using standard indices for each model. For the model including the sum of skinfold thickness, the root mean square error of approximation (RMSEA) lower bound was observed to be 0.000, and the standardized root mean squared residual (SRMR) value was 0.007, indicating a good fit. These fit indices were computed for all models to ensure a comprehensive evaluation of model fit. Variance inflation factors (VIF) were computed for all predictors to assess multicollinearity, with all VIF values being less than 2, suggesting low multicollinearity. Statistical significance was defined as a *p*‐value < 0.05. Stata software was used for all analyses. Results were interpreted using adjusted coefficients and 95% confidence intervals (CI).

## 3 Results

Table 1 shows differences in the descriptive characteristics of participants based on prenatal FA intake. In this study, 266 participants (11.39%) reported taking FA during pregnancy, while 2069 (88.61%) did not. Children of mothers who took FA were slightly younger (11.04 ± 0.62 years vs. 11.18 ± 0.68 years, *p* = 0.003), and their mothers were, on average, older (40.54 ± 4.64 years vs. 39.69 ± 4.95 years, *p* = 0.003). No significant differences were observed in biological sex distribution between the groups. However, a higher proportion of mothers who took FA had more than 14 years of education than those who did not consume FA supplements during their pregnancy (55.47% vs. 37.24%, *p* < 0.001). Other prenatal and birth characteristics showed no significant differences between the groups.

**TABLE 1:** Descriptive characteristics of the study population.

Variable	Folic acid intake during pregnancy		*p*
	Yes, 266 (11.39)	No, 2069 (88.61)	
	*N* (%) or mean ± SD		
	Median, IQR		
Demographic characteristics			
Age (years)	11.04 ± 0.62	11.18 ± 0.68	0.003^a^ ^,^ *
Biological sex			
Boy	142 (53.38)	1015 (49.06)	0.184^b^
Girl	124 (46.62)	1054 (50.94)	
Race			
Caucasians	263 (98.87)	2026 (97.92)	0.294^b^
Non‐Caucasians	3 (1.13)	43 (2.08)	
Maternal age (years)	40.54 ± 4.64	39.69 ± 4.95	0.003^a^ ^,^ *
Mothers educational level			
< 9 years	36 (13.58)	448 (21.98)	< 0.001^b^ ^,^ *
9–14 years	82 (30.94)	831 (40.78)	
> 14 years	147 (55.47)	759 (37.24)	
Prenatal and birth characteristics			
Gestational diabetes mellitus (GDM)			
Yes	8 (3.05)	50 (2.50)	0.592^b^
No	254 (96.95)	1952 (97.50)	
High blood pressure (HBP) during pregnancy			
Yes	13 (4.91)	61 (3.05)	0.109^b^
No	252 (95.09)	1943 (96.96)	
Smoking during pregnancy			
Yes	49 (18.42)	324 (15.66)	0.247^b^
No	217 (81.58)	1745 (84.34)	
Weight gain during pregnancy (kg)	14.61 ± 8.89	14.26 ± 8.79	0.543^a^
Small for gestational age			
AGA	214 (80.45)	1665 (80.47)	0.374^b^
SGA	37 (13.91)	247 (11.94)	
LGA	15 (5.64)	157 (7.59)	
Child characteristics			
BMI (kg/m^2^)	19.97 ± 3.61 19.28, 4.66	20.34 ± 3.87 19.28, 5.19	0.183^a^
Waist circumference (cm)	67.53 ± 8.98 65, 12	69.00 ± 9.74 67, 12.5	0.018^a^ ^,^ *
The sum of skinfold thickness (mm)	51.77 ± 20.89 48.75, 28.75	55.51 ± 23.13 52, 32.50	0.021^a^ ^,^ *
SBP (mmHg)	120.29 ± 13.21 120.50, 17.50	120.66 ± 13.21 120.50, 17.50	0.902^a^
DBP (mmHg)	68.85 ± 8.98 68, 12	69.98 ± 9.87 69, 12.50	0.119^a^
QUICKI	0.34 ± 0.03 0.34, 0.05	0.34 ± 0.03 0.34, 0.04	0.015^a^ ^,^ *
HOMA‐IR	2.51 ± 1.69 2.05, 1.95	2.72 ± 1.89 2.29, 1.79	0.015^a^ ^,^ *

^**a**^ Mann–Whitney *U* test.

^b^ Chi‐squared test.

* *p* < 0.05: statistically significant.

The difference in BMI between the groups was not statistically significant (*p* = 0.183). However, WC (median 65, IQR 12 vs. median 67, IQR 12.5, *p* = 0.018) and the sum of skinfold thickness (median 48.75, IQR 28.75 vs. median 52, IQR 32.50, *p* = 0.021) were significantly lower in the FA group.

No significant between‐group differences were observed in SBP (median 120.50, IQR 17.50 vs. median 120.50, IQR 17.50, *p* = 0.902) or DBP (median 68, IQR 12 vs. median 69, IQR 12.50, *p* = 0.119). In contrast, insulin resistance (HOMA‐IR) was significantly lower (median 2.05, IQR 1.95 vs. median 2.29, IQR 1.79, *p* = 0.015), while insulin sensitivity (QUICKI) was slightly higher (median 0.34, IQR 0.05 vs. median 0.34, IQR 0.04, *p* = 0.015) in the FA group compared to the group that did not use FA supplements.

The analysis of path coefficients and indirect effects using WC, skinfold thickness, or BMI as mediators revealed no significant direct effects of prenatal FA intake on SBP, DBP, QUICKI, or HOMA‐IR (Table 2; Tables S1 and S2). However, significant indirect effects were observed through WC, indicating that prenatal FA intake impacts SBP (coefficient = −0.69, *p* = 0.036) and DBP (coefficient = −0.36, *p* = 0.041), as well as QUICKI (coefficient = 0.0018, *p* = 0.044) and HOMA‐IR (coefficient = −0.1099, *p* = 0.047). Similarly, significant indirect effects through skinfold thickness were identified for SBP (coefficient = −0.52, 95% CI = [−1.03, −0.01], *p* = 0.046) and DBP (coefficient = −0.37, 95% CI = [−0.73, −0.01], *p* = 0.045). However, the indirect effects of FA intake on QUICKI and HOMA‐IR via skinfold thickness were insignificant (Table S1). Furthermore, when BMI was included as a mediator, no indirect effects of prenatal FA intake were observed on any of these metabolic outcomes (all *p*‐values > 0.05, Table S2).

**TABLE 2:** Direct and indirect effects of maternal folic acid intake on childhood metabolic outcomes in the models mediated by waist circumference.

Path	Coefficient	95% CI	*p*
Direct effects			
Folic acid intake → SBP	0.09	[−1.533, 1.71]	0.917
Folic acid intake → DBP	−0.58	[−1.70, 0.55]	0.313
Folic acid intake → QUICKI	0.0029	[−0.0010, 0.0068]	0.141
Folic acid intake → HOMA‐IR	−0.0546	[−0.2693, 0.1601]	0.618
Indirect effects			
Folic acid intake → waist circumference → SBP	−0.69	[−1.34, −0.04]	0.036^*^
Folic acid intake → waist circumference → DBP	−0.36	[−0.71, −0.01]	0.041^*^
Folic acid intake → waist circumference → QUICKI	0.0018	[0.00005, 0.0036]	0.044^*^
Folic acid intake → waist circumference → HOMA‐IR	−0.1099	[−0.2183, −0.0014]	0.047^*^

^*^ Statistical significance at *p* ≤ 0.05.

## 4 Discussion

Prenatal FA supplementation indirectly influences childhood metabolic outcomes like BP and insulin resistance through adiposity markers such as WC and skinfold thickness without direct effects. The variability in direct effects across models underscores the complexity of metabolic programming, likely influenced by different mediators and confounders. WC, as a marker of central adiposity, emerged as the strongest mediator due to its close link with visceral fat, which has high metabolic activity and is associated with hypertension, insulin resistance, and metabolic syndrome (Weiss and Kaufman 2008; Elks and Francis 2010). Skinfold thickness mediated the relationship between FA and BP, with no observed effect on insulin resistance, highlighting the role of subcutaneous fat in hypertension (Ruiz‐Alejos et al. 2020). This may be because subcutaneous fat, captured by skinfold thickness, influences BP through mechanical and vascular pathways. In contrast, visceral fat, reflected by WC, is more metabolically active and closely linked to insulin resistance. BMI, a general measure of body mass (including both fat and fat‐free mass), did not mediate these outcomes, highlighting the need to distinguish specific obesity markers in analyses (Du et al. 2023).

Emerging evidence highlights the critical role of maternal one‐carbon metabolism in the fetal programming of adiposity and metabolic health. The Pune Maternal Nutrition Study demonstrated that maternal vitamin B12 deficiency combined with high folate levels was associated with increased insulin resistance in offspring (Yajnik et al. 2008). Although maternal B12 status, a key component of one‐carbon metabolism, was not measured in our study, the observed indirect effects of FA via adiposity markers remain biologically plausible, consistent with broader micronutrient interactions. Supporting this, a recent twin cohort study found that specific maternal one‐carbon metabolites, such as dimethylglycine and choline, were associated with reduced fetal abdominal circumference and estimated fetal weight, highlighting the nuanced role of these pathways in growth regulation (Gong et al. 2023). Reviews by Finer et al. (2014) and Yajnik and Deshmukh (2012) further elucidate how alterations in maternal one‐carbon metabolism influence DNA methylation, adipocyte differentiation, and metabolic risk, providing a strong biological basis for these findings.

Clinical studies support these mechanistic pathways. For instance, in a Mexican birth cohort, González‐Ludlow et al. (2025) found that higher maternal folate concentrations during pregnancy were associated with increased neonatal adiposity, including both fat mass percentage and WC at birth. Randomized controlled trial evidence also supports a mechanistic link between FA intake and epigenetic programming. In the FASSTT trial, Irwin and coworkers demonstrated that continued FA supplementation into the second and third trimesters significantly altered DNA methylation patterns in cord blood, especially in genes related to growth and metabolism (Caffrey et al. 2018).

Epigenetic regulation may involve genes such as PPARG, GNAS, and RAS, which influence adipocyte differentiation, energy metabolism, and BP regulation. Methylation of PPARG, a master regulator of adipogenesis, could promote fat cell development and lipid storage, influencing offspring fat mass and distribution. Similarly, methylation changes in GNAS may impact adipocyte metabolism and insulin sensitivity, while RAS gene methylation has been associated with BP regulation and metabolic function (Abbas et al. 2024). Therefore, the lack of direct effects observed in our study may reflect these complex, multi‐step mechanistic pathways, in which prenatal FA influences intermediary phenotypes such as adiposity markers, which in turn impact childhood metabolic outcomes like BP and insulin resistance. This is consistent with epidemiological studies demonstrating strong associations between WC and BP across populations, as well as links between subscapular skinfold thickness and other metabolic risk factors (Ruiz‐Alejos et al. 2020).

These results are consistent with prior studies showing that prenatal micronutrient intake, particularly FA, shapes offspring metabolic health through adiposity‐related and epigenetic mechanisms (Jahan‐Mihan et al. 2024). Additionally, maternal folate status may affect the intrauterine metabolic environment; for instance, elevated maternal red blood cell folate levels in the second trimester have been associated with increased risk of gestational diabetes (Xie et al. 2019; Yang et al. 2021), a condition known to impact fetal adiposity and metabolic programming. Integrating these findings further strengthens the biological plausibility and relevance of our results within the current evidence base.

The results reinforce the potential biological pathways linking FA supplementation to metabolic outcomes via adiposity indicators. WC and skinfold thickness represent modifiable targets, providing a basis for public health interventions to optimize maternal nutrition during pregnancy to reduce the long‐term risk of childhood metabolic disorders. However, evidence of suboptimal adherence to prenatal supplementation guidelines (Bekele et al. 2024; Doru et al. 2023) along with widespread dietary diversity and micronutrient inadequacies among women in low‐ and middle‐income countries (Islam et al. 2024), highlights the gap between recommendations and real‐world practice. While the study employs rigorous statistical methods and robust control variables, its cross‐sectional design limits causal inference, and reliance on retrospective reporting introduces recall bias. Additionally, the focus on adiposity markers, though comprehensive, excludes other pathways like inflammation and genetic factors that warrant further exploration. Future longitudinal research is needed to address these limitations and confirm these findings across diverse populations.

## Author Contributions

Conceptualization: M.B., G.M., and B.E. Methodology: M.B., G.M., and B.E. Formal analysis: M.B. Investigation: G.M. and B.E. Resources: M.B., G.M., and B.E. Data curation: G.M. and Y.M. Writing – original draft preparation: Y.B. and M.B. Writing – review and editing: M.B., Y.B., E.K., G.M., and B.E. Supervision: G.M. and B.E. Project administration: G.M. and Y.M. All authors have read and agreed to the published version of the manuscript.

## Conflicts of Interest

The authors declare no conflicts of interest.

## Supporting information


**Table S1:** Direct and indirect effects of maternal folic acid intake on childhood metabolic outcomes in the models mediated by the sum of skinfold thickness.
**Table S2:** Direct and indirect effects of maternal folic acid intake on childhood metabolic outcomes in the models mediated by BMI.

## Data Availability

The authors have nothing to report.
