# Supplementary material for: Maternal Folic Acid Supplementation and Childhood Metabolic Health: Analyzing Direct and Indirect Effects
Source: Food Sci Nutr. 2025 Sep 16;13(9):e70959. doi: 10.1002/fsn3.70959 (PMC12441301; doi:10.1002/fsn3.70959)
Supplement: Supplementary file 1 — Table S1: Direct and indirect effects of maternal folic acid intake on childhood metabolic outcomes in the models mediated by the sum of skinfold thickness. Table S2: Direct and indirect effects of maternal folic acid intake on childhood metabolic outcomes in the models mediated by BMI. [file FSN3-13-e70959-s001.docx]

**Table S1: Direct and indirect effects of maternal folic acid intake on childhood metabolic outcomes in the models mediated by the sum of skinfold thickness**

| **Path** | **Coefficient** | **95% CI** | **p-value** |
| --- | --- | --- | --- |
| **Direct Effects** | | | |
| Folic Acid Intake → SBP | -0.14 | [-1.90, 1.62] | 0.874 |
| Folic Acid Intake → DBP | -0.60 | [-1.70, 0.49] | 0.278 |
| Folic Acid Intake → QUICKI | 0.0033 | [-0.0006, 0.0073] | 0.098 |
| Folic Acid Intake → HOMA-IR | -0.0847 | [-0.3089, 0.1393] | 0.458 |
| **Indirect Effects** | | | |
| Folic Acid Intake → Sum of Skinfolds → SBP | -0.52 | [-1.03, -0.01] | 0.046* |
| Folic Acid Intake → Sum of Skinfolds → DBP | -0.37 | [-0.73, -0.01] | 0.045* |
| Folic Acid Intake → Sum of Skinfolds → QUICKI | 0.0014 | [-0.00013, 0.0029] | 0.074 |
| Folic Acid Intake → Sum of Skinfolds → HOMA-IR | -0.0815 | [-0.1717, 0.0087] | 0.077 |

* Statistical significance at p ≤ 0.05.

**Table S2: Direct and indirect effects of maternal folic acid intake on childhood metabolic outcomes in the models mediated by BMI.**

| **Path** | **Coefficient** | **95% CI** | | **p-value** | |  |
| --- | --- | --- | --- | --- | --- | --- |
| **Direct Effects** | | | | | | |
| Folic Acid Intake → SBP | -0.05 | [-1.59, 1.49] | | 0.947 | |  |
| Folic Acid Intake → DBP | -0.68 | [-1.84, 0.48] | | 0.249 | |  |
| Folic Acid Intake → QUICKI | 0.0029 | [-0.0010, 0.0068] | | 0.080 | |  |
| Folic Acid Intake → HOMA-IR | -0.0546 | [-0.2693, 0.1601] | | 0.618 | |  |
| **Indirect Effects** | | | | | | |
| Folic Acid Intake → BMI → SBP | -0.59 | [-1.27, 0.10] | | 0.094 | |  |
| Folic Acid Intake → BMI → DBP | -0.27 | [-0.61, 0.06] | | 0.107 | |  |
| Folic Acid Intake → BMI → QUICKI | 0.0012 | [-0.0005, 0.0029] | | 0.152 | |  |
| Folic Acid Intake → BMI → HOMA-IR | -0.0858 | | [-0.1951, 0.0234] | | 0.124 | |
